# News and Events of the Week

**Published:** 1911-02-18

**Authors:** 


### News and Events of the Week.

Dr. Stenhouse, a London Missionary Society medical worker in Peking, has gone, says the *Times* correspondent, with two other Missionary doctors to assist in plague prevention work in Kharbin. Dr. Gibb, another London Missionary Society doctor, who had preceded them by a fortnight, sends word that he is well.

The 32nd anniversary dinner of the Society of Public Analysts was given last Friday, Mr. E. W. Voelcker, the President, in the chair.

The late Lord Winterstoke, whose generous gift of £5,000 towards the extension of the Bristol General Hospital so closely preceded his decease, was one of the first to take an active interest in deep-sea fishermen. The kindly gift which he shared in bestowing more than a quarter of a century back was a liberal supply of Wills' tobacco for the fishermen of the Dogger Bank, a fact which was only made public when fuller accounts of the fishing life there began to appear in the late 'eighties.

Dr. Alan Leslie Fielding, of Ottery St. Mary, Devon, died in an hotel in Villiers Street, Strand, on Saturday last. Dr. Fielding, who registered L.R.C.P.Edin., L.R.C.S.Edin. in 1902, and who was 35 years of age, missed his train on Friday night, February 10, and applied for a room at the hotel. On Saturday morning his body was found, fully dressed, on the bed. Dr. Fielding leaves a widow and two children.

Commenting on the satisfaction felt in Canada on the official announcement of the Duke of Connaught's appointment as Governor-General, the *Times* correspondent of Ottawa says: Especially is the announcement of the Duke's appointment welcomed by friends of the St. John Ambulance movement, which since the organisation of the Canadian branch by Mr. Harold Boulton a year ago has made great strides. It is felt that the presence in Canada of the Grand Prior of the Order of St. John will give considerable impetus to the work.

At the Institute of Hygiene an experienced *chef* is giving free demonstrations with the object of giving information or advice on either any branch of cooking or the best method of cooking any particular dish. For years past a qualified medical man has been giving information at the Institute of Hygiene on foods. The *chef* will be in attendance daily at 34 Devonshire Street, Harley Street, W., from 11 to 1 and from 3 to 5.

A translation of "Cymbeline" in French verse, adapted for stage purposes, has just been brought out by Dr. Henri Fauvel, a doctor-poet of Normandy, who was a leading medical practitioner in Havre for many years. He has dedicated this Shakespearean translation, which is expected (so states the preface to the work) to be staged shortly, to Mr. Algernon Warren, who was formerly connected with the old-established firm, now succeeded by Messrs. Evans, Gadd and Co., of A. and J. Warren, wholesale druggists, Bristol, and reviewed a French translation of Tennyson's "Maud" by Dr. Fauvel, which led to an intimacy. When connected with the Havre Hospital Dr. Fauvel found his acquired knowledge of English of much service in interpreting and dealing with the symptoms of numbers of British seamen who were constantly being brought there.

The Peninsular and Oriental Company's steam-yacht "Vectis" left Marseilles on February 9 on the first of her spring cruises. The itinerary includes Greece, Syria, the Holy Land, Egypt, Sicily, and Naples.

An inquest on the body of Mr. Gerald George Hodgson, M.R.C.S., L.S.A., of Stoneleigh, Reigate, who for many years was in practice at Brighton, was held at Hillingdon last Tuesday. It was found that Dr. Hodgson had taken an overdose of morphine. The jury returned a verdict of "Death by misadventure."

The Royal Commission on Vivisection held its fifty-second meeting last week. All the members (Mr. Ram in the chair, Colonel Lockwood, Sir William Church, Sir William Collins, Sir John McFadyean, Sir M. D. Chalmers, Dr. Gaskell, Dr. Wilson) and the secretary were present. The discussion of the report was proceeded with.

The Lannelongue Prize, founded last year by Professor Lannelongue—a gold medal, with a sum of £200—to be awarded to the person who had contributed most to the progress of surgery in the ten years before the date of the award, has been presented to Sir Victor Horsley. The prize is open to surgeons of all nations, and is awarded every five years.

The committee of the Athenæum Club has elected among others, Surgeon-General Sir Alfred Keogh, K.C.B., LL.D., Rector of the Imperial College of Science and Technology, according to the provisions of rule ii. of the club, which empowers the annual election by the committee of a certain number of persons "of distinguished eminence in science, literature, the arts, or for public services."

The medical practitioners of Liverpool have held a meeting to protest against the scheme recently proposed by the Education Committee of Liverpool under which children found to be defective may be treated at the local hospitals at fees lower than those generally charged by private practitioners. Resolutions were passed pledging those present to support the medical staffs of the local hospitals in any objection they might make to the scheme.

Dr. Achille Kelsch, member of the French Academy of Medicine and an eminent officer of the French Army Medical Service, has died in his 71st year. Dr. Kelsch devoted much study to epidemics, and it was as Professor of Epidemiology that after two years' service in Algeria he was appointed to the Medical School of Val de Grâce in the early eighties. While in Africa he had investigated the diseases peculiar to warm climates, and published the result of his researches soon after his return from Algeria. Dr. Kelsch was the founder and director of the Vaccination Institute of the Academy of Medicine.

The following are the arrangements for next week at the West London Postgraduate College: February 20 and 23, at 10 a.m., Demonstration by Surgical Registrar; February 20, at 12 noon, Pathological Demonstration; February 21, at 11.30 a.m., Demonstration of Minor Operations; February 22, at 10 a.m., Gynæcological Demonstration; February 23, at 12.15 p.m., Lecture, Dr. Grainger

Stewart, Practical Medicine. Lectures at 5 P.M. : February 20, Mr. Etherington Smith, Clinical Lecture II.; February 21, Dr. Pritchard, Practical Medicine; February 22, Mr. Lloyd Williams, "Oral Conditions in their Relation to General Conditions of Health and Disease"; February 23, Mr. Baldwin, Practical Surgery; February 24, Mr. Rickard Lloyd, Anæsthetics.

The following are the arrangements for next week at the Medical Graduates' College and Polyclinic, 22 Chenies Street, W.C. : Monday, February 20, at 4 P.M., Dr. J. M. H. MacLeod, Clinique (Skin); at 5.15 P.M., Dr. H. D. Rolleston, "Some Aspects of Lymphadenoma." Tuesday, at 4 P.M., Dr. J. J. Perkins, Clinique (Med.); at 5.15 P.M., Mr. Arthur Edmunds, "The Surgical Treatment of Tumours of the Breast." Wednesday, at 4 P.M., Mr. F. C. Wallis, Clinique (Surg.); at 5.15 P.M., Dr. W. H. Kelson, "Some Common Affections of the Ear" (illustrative cases). Thursday, at 4 P.M., Dr. Theodore Thompson, Clinique (Med.); at 5.15 P.M., Dr. William Hill, "Demonstration of a New Method, combining Direct and Indirect Gastroscopy." Friday, at 4 P.M., Mr. Ernest Clarke, Clinique (Eye).

